# Novel Approach for Cardioprotection: In Situ Targeting of Metformin via Conductive Hydrogel System

**DOI:** 10.3390/polym16152226

**Published:** 2024-08-05

**Authors:** Ying Tan, Jierong Li, Yali Nie, Zhi Zheng

**Affiliations:** Hunan Provincial Key Laboratory of Multi-Omics and Artificial Intelligence of Cardiovascular Diseases & Institute of Cardiovascular Disease & Department of Cardiology, Hengyang Medical School, University of South China, Hengyang 421001, China

**Keywords:** ischemia/reperfusion, conductive hydrogel, metformin, mitochondria

## Abstract

Ischemia/reperfusion (I/R) injury following myocardial infarction is a major cause of cardiomyocyte death and impaired cardiac function. Although clinical data show that metformin is effective in repairing cardiac I/R injury, its efficacy is hindered by non-specific targeting during administration, a short half-life, frequent dosing, and potential adverse effects on the liver and kidneys. In recent years, injectable hydrogels have shown substantial potential in overcoming drug delivery challenges and treating myocardial infarction. To this end, we developed a natural polymer hydrogel system comprising methacryloylated chitosan and methacryloylated gelatin modified with polyaniline conductive derivatives. In vitro studies demonstrated that the optimized hydrogel exhibited excellent injectability, biocompatibility, biodegradability, suitable mechanical properties, and electrical conductivity. Incorporating metformin into this hydrogel significantly extended the administration cycle, mitigated mitochondrial damage, decreased abnormal ROS production, and enhanced cardiomyocyte function. Animal experiments indicated that the metformin/hydrogel system reduced arrhythmia incidence, infarct size, and improved cardiac mitochondrial and overall cardiac function, promoting myocardial repair in I/R injury. Overall, the metformin-loaded conductive hydrogel system effectively mitigates mitochondrial oxidative damage and improves cardiomyocyte function, thereby offering a theoretical foundation for the potential application of metformin in cardioprotection.

## 1. Introduction

Acute myocardial infarction (AMI) is a leading cause of increased global mortality rates in cardiovascular disease [1,2]. Currently, the rapid and safe restoration of blood supply to ischemic myocardium is recognized as the optimal and most effective treatment for AMI [3]. Nevertheless, revascularization surgery can exacerbate heart muscle damage, leading to a secondary insult known as ischemia/reperfusion (I/R) injury [4]. Investigating measures and mechanisms to prevent I/R injury is crucial for improving the outlook and survival rates of cardiovascular disease patients [5,6]. Multiple medications have shown cardioprotective properties, reducing the negative impacts of ischemic therapy on blood vessels [7,8]. Among these drugs, post-treatment with metformin (metf) has demonstrated a protective effect against myocardial I/R injury, attracting significant attention from researchers [9,10,11]. Moreover, research has indicated that the use of metformin is associated with reduced cardiovascular mortality and incidence rates in patients with type 2 diabetes compared to other antidiabetic medications [12,13]. The primary cause of I/R injury is the inadequate supply of oxygen and nutrients to cellular mitochondria, resulting in their dysfunction. This dysfunction leads to the generation of a significant amount of reactive oxygen species (ROS) and activates the intracellular caspase signaling pathway, ultimately leading to apoptosis [11,14,15]. The accumulation of apoptotic cardiomyocytes (CMs) contributes to fibrosis in the infarcted area, leading to myocardial failure. Thus, the potential mechanism through which metf improves I/R injury may be linked to the regulation of mitochondrial function in CMs. However, metf administration has certain limitations, such as a short half-life, frequent dosing, and the potential development of tolerance and reduced efficacy with long-term use [16,17]. In addition, metf often causes gastrointestinal side effects like nausea, vomiting, and diarrhea, and prolonged high-dose usage may harm kidney function. Therefore, developing an in situ targeted sustained-release formulation of metf could be a promising strategy to improve bioavailability, decrease the dosing frequency, and effectively manage adverse drug reactions.

In recent years, injectable hydrogels responsive to various biological and chemical signals have been utilized in a minimally invasive manner to enable precise loading and an on-demand release of bioactive therapeutic agents, resulting in effective treatment outcomes [18,19,20,21]. Furthermore, the injection of conductive hydrogels into the infarcted area can facilitate electrical signaling during the systolic–diastolic mechanical cycle, while also providing mechanical support to the damaged myocardium [20,22,23]. However, many current hydrogels used for myocardial repair are composed of weakly conducting or non-conducting materials, which may adversely affect the repair results. Therefore, the selection of suitable materials for constructing hydrogel carriers with excellent performance is crucial in significantly improving myocardial repair in infarcted tissues [24,25,26]. Natural biomaterials possess modifiable sites, excellent biocompatibility, and degradability [27,28]. Chitosan, derived from the deacetylation of the natural polysaccharide chitin, exhibits advantages such as biodegradability, biocompatibility, and suitability for medical applications [29]. The abundance of amino and hydroxyl groups on chitosan enables its modification for cross-linking and the formation of hydrogel networks. However, single-gel networks feature large pore structures and poor mechanical properties, potentially resulting in the rapid release of encapsulated drugs and a shorter gel lifespan in vivo [30]. By forming multiple gel networks, a denser network structure is achieved, enhancing the mechanical properties of the gel. Gelatin, a derivative of collagen through partial hydrolysis, possesses outstanding biocompatibility and degradability and notably contains RGD peptide sequences that serve as cell adhesion sites [22]. Conductive polymer modification of gelatin can confer electrical conductivity, enabling the cross-linking of modified gelatin with chitosan to form a hydrogel network that shows promise as a drug carrier [25].

Based on the specific site of myocardial infarction and the blind-end effect of the drug vasculature system, we developed a hydrogel system by cross-linking methacryloylated chitosan (CSMA) and conductive derivative-modified methacryloylated gelatin (Pam-GelMA) through UV light irradiation. To optimally match myocardial tissue, the optimized hydrogel formulation exhibits excellent biocompatibility, biodegradability, suitable mechanical properties, and electrical conductivity. In vitro and in vivo studies showed that the slow in situ long-term release of metformin from this hydrogel depleted abnormal ROS levels in cardiomyocytes, ameliorated mitochondrial damage, and promoted CM activity. Additionally, the conductive hydrogel improved arrhythmia by connecting the infarcted and normal regions for electrical signal conduction, promoting cardiac function and enhancing myocardial repair. This study elucidates a metformin/hydrogel system for the treatment of I/R injury, providing a theoretical reference for understanding the cardioprotective and therapeutic mechanisms of metformin.

## 2. Materials and Methods

### 2.1. Materials

Gelatin, chitosan, methacrylic anhydride, N-(3-aminopropyl)methacrylamide hydrochloride, N-hydroxysuccinimide (NHS), 1-(3-dimethylaminopropyl)-3-ethylcarbodiimide hydrochloride (EDC·HCl), 3-amino-4-methoxybenzoicacid, camphorsulfonic acid, ammonium persulfate (APS), diphenyl(2,4,6-trimethylbenzoyl)phosphine oxide, trimethylamine, and H_2_O_2_ were purchased from Aladdin (Hangzhou, China). Metformin mesylate was supplied by Selleck (Houston, TX, USA). The dihydroethidium (DHE) assay kit, TUNEL kit, MTT test kit, and live/dead cell staining kit were obtained from Solarbio (Beijing, China).

### 2.2. Synthesis of CSMA and Pam-GelMA

For the preparation of CSMA, a 1% wt chitosan solution was created by dissolving chitosan powder in an acetic acid solution. The chitosan solution was then mixed with methacrylated anhydride, and the reaction was conducted at 60 °C for 6 h. The resulting solution underwent dialysis for a minimum of 5 days and was subsequently freeze-dried to obtain the CSMA product. For the preparation of Pam-GelMA, a mixture of gelatin, 3-amino-4-methoxybenzoicacid, and (±)-10-camphorsulfonic acid in 100 mL of ultrapure water was stirred for 30 min. Afterwards, APS was introduced and the reaction was left to continue for a duration of 12 h. The mixture was dialyzed in pure water for a minimum of 3 days before being freeze-dried. Pam–Gelatin was first dissolved in water and then mixed with 0.2 g of EDC and 0.12 g of NHS at a pH of 4.5 to activate the carboxyl groups. This activation process was maintained for 4 h. N-(3-aminopropyl)methacrylamide hydrochloride was then added to the reaction mixture and allowed to react for 24 h. Once the reaction was complete, the mixture was placed in an MD44 dialysis bag and dialyzed for at least 3 days. After dialysis, the Pam-GelMA product was frozen and lyophilized to produce the final product. FT-IR (Bruker, Wurzbach, Germany) and ^1^H NMR (Bruker, 400 MHz) were employed to examine the molecular compositions of CSMA and Pam-GelMA.

### 2.3. Cytotoxicity Assay

The absorbance value of exogenous MTT, reduced by succinate dehydrogenase in living cell mitochondria, was measured at 490 nm using an enzyme immunoassay detector. This measurement serves as an indirect indicator of the number of living cells. The formation of MTT crystals is directly proportional to the cell count within a specific range.

### 2.4. Rheological Testing of Hydrogels

Cylindrical hydrogel specimens, measuring 1 cm in diameter and 1 cm in height, were analyzed for G′ and G″ using a rotational rheometer from Malvern, UK. Time sweep tests were conducted over a 0–20 min period, and frequency sweep tests ranged from 0.1 to 10 Hz.

### 2.5. Live/Dead Staining

CMs were cultured in 96-well plates using a 10 μL/well hydrogel precursor solution. After 12 h, CM viability was assessed through the MTT assay. To differentiate between viable and non-viable CMs, Calcein–AM and PI staining were carried out according to the manufacturer’s instructions. After rinsing with PBS, CM viability and growth were observed using a laser confocal microscope. Viable cells exhibited green fluorescence at 490 nm from Calcein–AM, while dead cells were identified by red fluorescence at 545 nm from PI.

### 2.6. Detection of Metf Release

The metf-containing hydrogel was loaded into a dialysis bag and immersed in PBS, then gently stirred in a light-protected environment. Throughout the experiment, 1 mL samples were collected at specific time intervals and replaced with fresh PBS. UV-Vis spectroscopy was employed to analyze the absorption of the metf solution at 233 nm. By comparing the release pattern of metf from the hydrogel with the metf calibration curve, the release rate was calculated.

### 2.7. JC-1 Staining

Cultured CMs were collected under various experimental conditions and washed with PBS to remove any solution and contaminants. Subsequently, the cells were mixed with 1 mL of JC-1 staining solution, ensuring thorough mixing for uniform coverage. The cells were then incubated at 37 °C for 20 min to allow the JC-1 dye to interact effectively with the mitochondrial membrane potential, producing accurate fluorescence signals. During incubation, the excess staining solution was removed, and the cells underwent two washes with JC-1 staining buffer to ensure specificity. Cell viability was maintained by adding a fresh cell culture medium post-washing. The stained cells were then analyzed using a fluorescence microscope with excitation light at 490 nm and emission light at 530 nm to visualize and quantify mitochondrial membrane potential changes in the cultured CMs.

### 2.8. ROS Detection

DHE was used to evaluate the levels of superoxide anion and ROS within CMs intracellularly. CMs were grown in 48-well dishes and exposed to oxidative stress damage through the introduction of 0.1 mM hydrogen peroxide. Subsequently, the CMs were treated with a hydrogel precursor and then incubated for 12 h. Staining of the CMs was performed using DHE. The specimens were observed with a Nikon Ti A1 fluorescence microscope (Ti-E+A1 Si, Tokyo, Japan), and ImageJ (Plus 6.0) software was used to measure the fluorescence levels of DHE.

### 2.9. Establishment of Rat Model

This study obtained a group of 60 male SD rats weighing 220 ± 30 g from HUNAN SJA LABORATORY ANIMAL CO., LTD. (Changsha, China). Seven rats died during the experimental procedures and follow-up period. Myocardial infarction was induced by ligating the left anterior descending (LAD) artery near its origin, following an established protocol [20,21]. Subsequently, 100 μL of hydrogel was administered to five separate sections in the damaged area, with each section receiving 20 μL. A hydrogel patch containing 100 μL of hydrogel was applied directly to the surface of the damaged heart muscle within the pericardial sac. The Experimental Animal Ethics Committee of the University of South China, Hunan, People’s Republic of China approved all animal-related experimental protocols (approval 4304079008946), in accordance with guidelines set by the National Institutes of Health and the University of South China for the ethical treatment and utilization of animals in scientific studies.

### 2.10. Echocardiographic Analysis

All rat groups underwent cardiac function evaluation at various time points after surgery (7, 14, 21, and 28 days) using the VisualSonics VEVO2100 (Toronto, ON, Canada) small animal ultrasound machine. Cardiac function was assessed using 2D transthoracic ECHO (VisualSonics, Toronto, ON, Canada) in the M-mode while the rats were under isoflurane anesthesia. The ECHO assessment for each rat involved measuring the LVIDS, LVIDD, LVFS, and LVEF.

### 2.11. Histological Analysis

Following euthanasia, the rat hearts were extracted and prepared for further examination. After being submerged in a 4% paraformaldehyde solution, the hearts were then encased in paraffin and cut into sections. Masson and H&E staining were used to assess the infarct and fibrosis areas. In addition, TUNEL staining identified apoptotic cells, and α-actinin staining highlighted the structural framework of the heart. Fluorescence microscopy was used to detect and measure apoptotic and healthy cells in the injured tissue.

### 2.12. Statistical Analysis

GraphPad Prism 7 software was used for statistical analyses. The data were expressed as the mean ± standard deviation (n = 3). A Student’s *t*-test was used to assess statistical significance in the comparison of two groups, with significance thresholds established at * *p* < 0.05, ** *p* < 0.01, and *** *p* < 0.001.

## 3. Results and Discussion

### 3.1. Preparation of CSMA/Pam-GelMA Hydrogels

**Figure 1:**
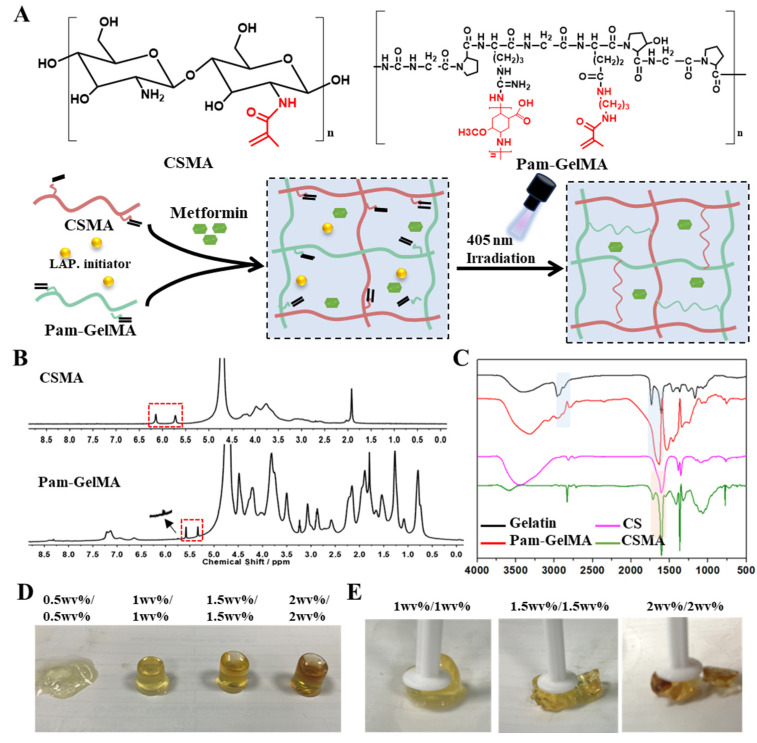
Preparation of CSMA/Pam-GelMA hydrogels. (**A**) Preparation process of CSMA/Pam-GelMA hydrogels. (**B**) ^1^H NMR spectra of CSMA and Pam-GelMA. (**C**) FT-IR spectra of CS, CSMA, gelatin, and Pam-GelMA. (**D**) Photographs of different formulations forming hydrogels. (**E**) Photographs of different formulated hydrogels subjected to equal pressure.

Both chitosan and gelatin are natural biodegradable materials known for their good biocompatibility, making them suitable for various medical and biomedical applications. Gelatin is primarily degraded in the body through the action of collagenase and hydrolysis, ultimately breaking down effectively into small peptide chains and amino acids, which are then absorbed by the body. Chitosan degrades in the human body through the following mechanisms: (i) Enzymatic degradation: Chitosan is primarily degraded by chitosanases in the body, with higher activity in the liver and other metabolically active organs. (ii) Acidic environment: Chitosan can also be partially degraded in acidic microenvironments. Although human blood is generally neutral, localized sites of inflammation or infection may lower the pH, promoting chitosan degradation. (iii) Role of the immune system: The immune system can also contribute to chitosan degradation. Immune cells such as macrophages can phagocytose and decompose chitosan, which is further degraded in the acidic environment of lysosomes. The final degradation products of chitosan are mainly N-acetylglucosamine and glucosamine, which can be further metabolized and utilized in the body or excreted through urine. Overall, chitosan degradation in the bloodstream is a complex process involving the synergistic action of multiple enzymes and the immune system. In addition, their exceptional water absorption properties have contributed to their popularity in moisturization, drug delivery, and wound dressing. A straightforward and reproducible UV irradiation-based method was utilized for the successful preparation of a natural double-cross-linked hydrogel, derived from methacrylamidated chitosan and methacrylamidated gelatin (Figure 1A). To impart electrical conductivity to the hydrogel, we introduced a modification of the gelatin with the polyaniline derivative 3-amino-4-methoxybenzoicacid, resulting in the formation of Pam–Gelatin. The successful synthesis of Pam–Gelatin was confirmed through the observation of characteristic peaks at approximately 5.75 ppm in the ^1^H NMR spectroscopy. In addition, the MA characteristic peaks at 5.33 ppm and 5.57 ppm confirmed the successful synthesis of Pam-GelMA. Integration of the peak areas indicated that the modification rate was approximately 6.8% for Pam and over 80% for MA (Figure 1B). Similarly, characteristic peaks at 5.72 ppm and 6.14 ppm in the ^1^H NMR spectra validated the synthesis of CSMA (Figure 1B). Further confirmation of the successful synthesis of CSMA and Pam-GelMA was obtained through FT-IR spectra analysis (Figure 1C).

The preparation of hydrogels involves using different formulations of CSMA and Pam-GelMA, which are then exposed to 405 nm ultraviolet light for gel formation. For instance, a hydrogel with 1.5 wv% CSMA and 1.5 wv% Pam-GelMA is dissolved in 500 mL of physiological saline solution. The mixture is then exposed to 405 nm ultraviolet light for 10 s, resulting in immediate gel formation.

### 3.2. Characterization of CSMA/Pam-GelMA Hydrogels

**Figure 2:**
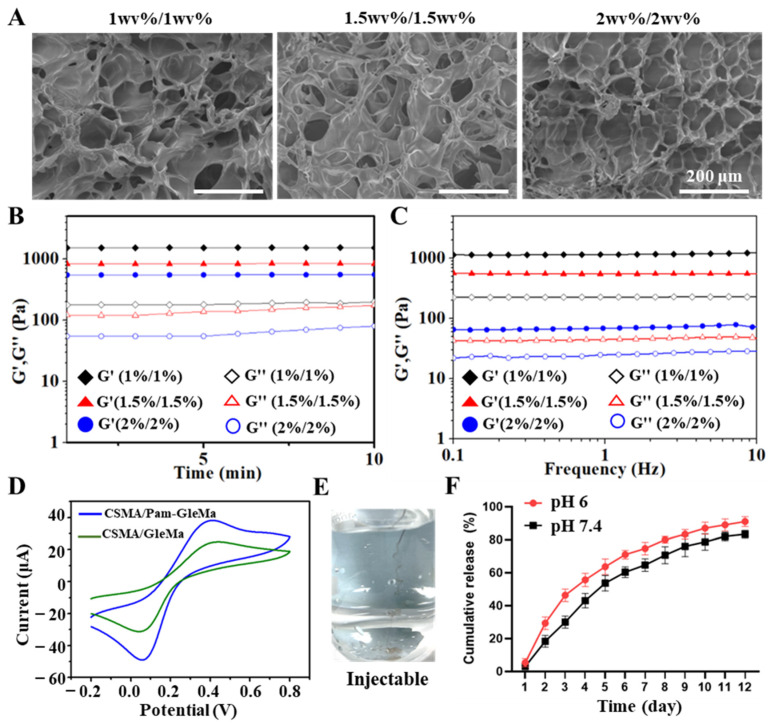
Characterization of CSMA/Pam-GelMA hydrogels. (**A**) SEM images of CSMA/Pam-GelMA hydrogels with different formulations. (**B**) Rheological time-scanning plots of CSMA/Pam-GelMA hydrogels. (**C**) Rheological frequency-scanning plots of CSMA/Pam-GelMA hydrogels. (**D**) CV curve of CSMA/Pam-GelMA hydrogels. (**E**) Photograph of injectability of CSMA/Pam-GelMA hydrogel. (**F**) Metf release cycle at pH 6.0 and pH 7.4.

Hydrogels with weaker mechanical strength may not adequately support the infarcted myocardial wall, while those with stronger mechanical properties could potentially form a dense gel network that hinders drug release. Hydrogels of different formulations (0.5 wv%/0.5 wv%, 1 wv%/1 wv%, 1.5 wv%/1.5 wv%, and 2 wv%/2 wv%) were tested to determine their flexibility and mechanical properties. The 0.5%/0.5% hydrogel failed to form due to its poor mechanical performance, while the 1.5%/1.5% and 2%/2% hydrogels exhibited excessive mechanical strength, leading to a tendency for brittle fracture (Figure 1D). On the other hand, the 1%/1% hydrogel displayed superior performance in terms of both flexibility and mechanical strength, making it more suitable for both in vitro and in vivo research (Figure 1E). Subsequently, the pore size of these three hydrogel formulations was examined, revealing that all three had uniformly distributed pores, with smaller pore sizes observed with increasing concentration (Figure 2A). The storage modulus (G′) and loss modulus (G″) of hydrogels with concentrations of 1%/1%, 1.5%/1.5%, and 2%/2% were examined using a rotational rheometer in a time-scan mode lasting 10 min. The results revealed that the modulus increased with higher concentrations, with the 2%/2% hydrogels showing the highest mechanical strength (Figure 2B). Throughout the frequency scanning process, it was observed that the G′ values of all hydrogel samples were consistently higher than the G″ values within the 0.1 to 10 Hz range, indicating the presence of a cross-linked network characteristic of an elastic solid (Figure 2C).

Cyclic voltammetry (CV) is a method used to assess the electrical conductivity of hydrogels. The CV curves of CSMA/Pam-GelMA hydrogels, when modified with Pam, showed significant widening, suggesting a stronger capacitive ability of CSMA/Pam-GelMA hydrogels. Conversely, the smaller closed region of the CV curves indicated a relatively weaker capacitive ability of CSMA/GelMA hydrogels (Figure 2D). Moreover, the 1%/1% CSMA/Pam-GelMA exhibited excellent injectability, making it suitable for intra-tissue injections (Figure 2E).

Metformin is an oral drug commonly used to treat type 2 diabetes. In recent years, it has also been found to have positive effects on conditions such as cardiovascular disease, fatty liver, and tumors. In much of the literature and daily treatment, metformin is primarily delivered orally and intraperitoneally, but this raises the issue of the first-pass effect, whereby some of the drug enters the circulation in reduced amounts after metabolism through the intestinal lumen, intestinal wall, or liver. Due to the specificity of myocardial infarction sites, direct injection at the infarction site requires multiple doses to ensure safety, but each opening of the chest cavity significantly increases the risk of death in rats. Therefore, we designed a method to encapsulate metformin in a hydrogel, allowing the hydrogel to encapsulate a large dose of metformin for in situ passive targeting, which improves the drug’s bioavailability and overcomes the need for multiple administrations.

A pH of 7.5 was used as a normal control, while the pH in the myocardial infarction microenvironment was slightly acidic, approximately 6.0. To simulate the pH of the infarction microenvironment, two experiments with different pH values were conducted to observe the differences in the results. In vitro experiments examined the release of metf from a 1%/1% hydrogel. Metf was incorporated into the hydrogels, and its release was monitored by measuring UV absorbance at 233 nm in 1 mL samples taken at specific time intervals. The findings revealed that more than 90% of metf was released within 12 days at pH 6.0, indicating a faster release rate compared to pH 7.4 (Figure 2F). These findings imply that the 1%/1% hydrogels possess favorable mechanical properties, flexibility, pore size, and drug release kinetics for further potential in vivo studies.

### 3.3. Effect of Metf/Hydrogel on ROS-Induced CM Activity

**Figure 3:**
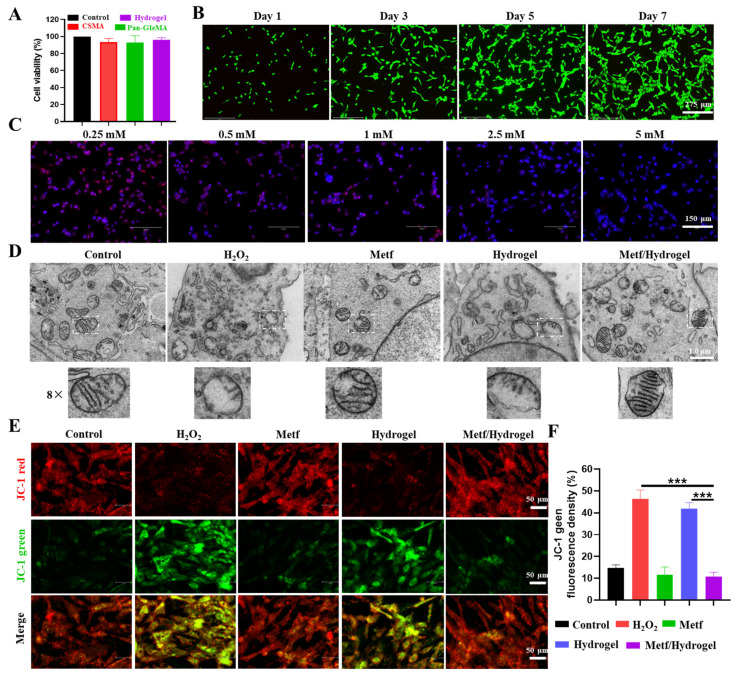
Effect of metf/hydrogel on CM activity. (**A**) MTT assay to detect effect of different components on CM cytocompatibility. (**B**) Live/dead cell assay to assess effect of hydrogel on CM activity in culture for one week. (**C**) DHE staining kit to detect CM oxidative damage. (**D**) Representative TEM images of cardiac muscle mitochondria from each experimental group were examined. (**E**) Fluorescence images of JC-1 were taken to evaluate membrane potential of mitochondria in CM in all groups. (**F**) Quantitative fluorescence analyses are from JC-1. (n = 3, *** *p* < 0.001).

Hydrogels composed of natural biomaterials exhibit excellent cytocompatibility and biodegradability. The cytocompatibility of these hydrogels was evaluated using a CCK-8 assay and live/dead cell staining to study the effects of different compositions on cell viability. The results showed that the various hydrogel extracts had non-significant effects on cell viability, suggesting that the hydrogel was non-toxic (Figure 3A). Furthermore, cytocompatibility was assessed after one week of cell culture using live/dead cell staining kits. Calmodulin AM and PI staining showed minimal cell death throughout the cell culture period, and the cells remained highly viable even after 7 days of culture (Figure 3B).

Myocardial damage post-MI is primarily due to malfunctioning mitochondria and ROS generation. ROS-triggered cell death releases cellular content, attracting inflammatory cells to the infarcted area and exacerbating the inflammatory response. Timely intervention is crucial in preventing cell death and the inflammatory cascade after infarction by targeting mitochondrial dysfunction and reducing ROS production. Metf has been suggested to have antioxidant properties in myocardial mitochondria, potentially delaying oxidative stress-induced mitochondrial damage. However, the effectiveness of metf may be influenced by suboptimal or excessive concentrations, leading to either inefficiency or adverse effects. To determine the optimal concentration of metf for regulating intracellular ROS levels in CMs, oxidatively damaged CMs were treated with 0.25–5 mM metf. DHE staining was used to assess the presence of superoxide anions and hydroxyl radicals in cells. Six hours after H_2_O_2_ treatment, the control group CMs showed elevated intracellular ROS levels, as evidenced by intense red fluorescence from DHE staining. Upon the addition of metf, intracellular DHE intensity gradually decreased with higher metf concentrations, indicating a beneficial antioxidant effect at 5 mM (Figure 3C). Consequently, 5 mM metf was chosen for subsequent experimental investigations.

After oxidative damage, the cristae of mitochondria in CMs transition from a reticular to a fragmented structure. Observation using electron microscopy revealed that groups treated with metf alone or in combination with hydrogel exhibited a protective effect against mitochondrial cristae damage and improved cellular membrane integrity, in contrast to the substantial mitochondrial network fragmentation induced by H_2_O_2_ (Figure 3D). Furthermore, the impact of hydrogel samples on mitochondrial membrane potential changes after ROS-induced CM damage was evaluated using the JC-1 kit. The results indicated a notable decrease in green fluorescence intensity in CMs treated with metf and metf/hydrogel, while the H_2_O_2_ group exhibited elevated green fluorescence levels, suggesting a potent mitochondrial antioxidant effect of metf (Figure 3E,F). In summary, the metf/hydrogel combination effectively reduced abnormal ROS generation by mitigating mitochondrial cristae damage and enhancing mitochondrial membrane potential, ultimately leading to improved CM function.

### 3.4. Effect of Metf/Hydrogel on Heart Structure and Function in Rats with MI

**Figure 4:**
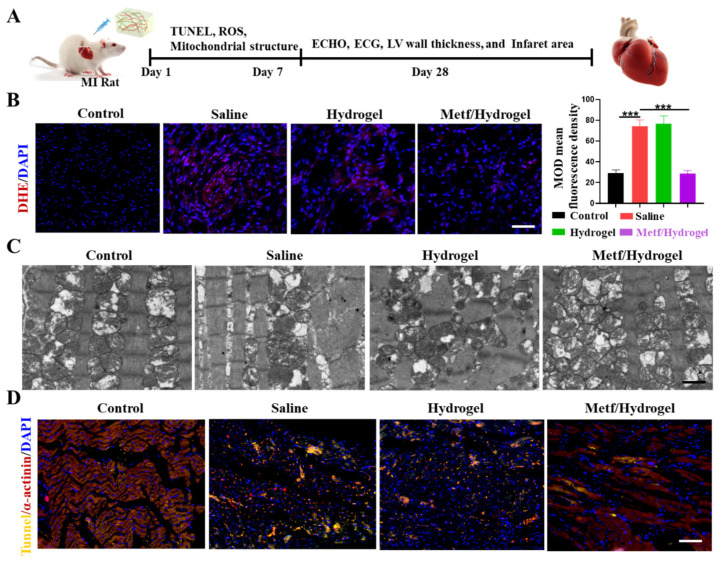
Effect of metf/hydrogel on cellular activity in infarcted region. (**A**) DHE staining of infarcted areas in all groups. (**B**) ROS staining of infarcted region in all groups. (**C**) Fluorescence quantification of DHE staining in all groups. (**D**) Bioelectron microscopy of myocardial tissue in infarcted regions of all groups (scale bar: 2 μm). (n = 3, *** *p* < 0.001).

Compared to high-strength materials, softer hydrogels offer advantages such as minimizing surgical trauma, facilitating application to the area of MI, and simplifying surgical procedures while reducing risks. This study aimed to explore the therapeutic benefits of metf/hydrogel on MI, with four distinct experimental groups: normal, saline, blank hydrogel, and metf/hydrogel. Figure 4A illustrates the experimental timeline of metf/hydrogel application in MI rats. To account for the in situ release of metf and the potential adverse effects of high doses, metf was encapsulated in 100 mL of hydrogel at a concentration of 5 mM. Levels of ROS in the infarcted region were measured using DHE staining after 5 days of metf/hydrogel treatment to analyze its impact on ROS clearance in the initial phases of MI. The results showed a notable reduction in DHE signals in the metf/hydrogel group compared to the saline and blank hydrogel groups, suggesting the successful removal of ROS in the area affected by MI (Figure 4B). Furthermore, an examination of myocardial mitochondrial structure using bio-TEM revealed that the metf/hydrogel group effectively ameliorated mitochondrial cristae damage compared to the blank hydrogel group, further demonstrating the efficacy of metf in mitigating mitochondrial damage (Figure 4C). TUNEL staining on the 5th day of treatment demonstrated that the metf/hydrogel group had the lowest CM apoptosis rate, highlighting its therapeutic advantage (Figure 4D).

**Figure 5:**
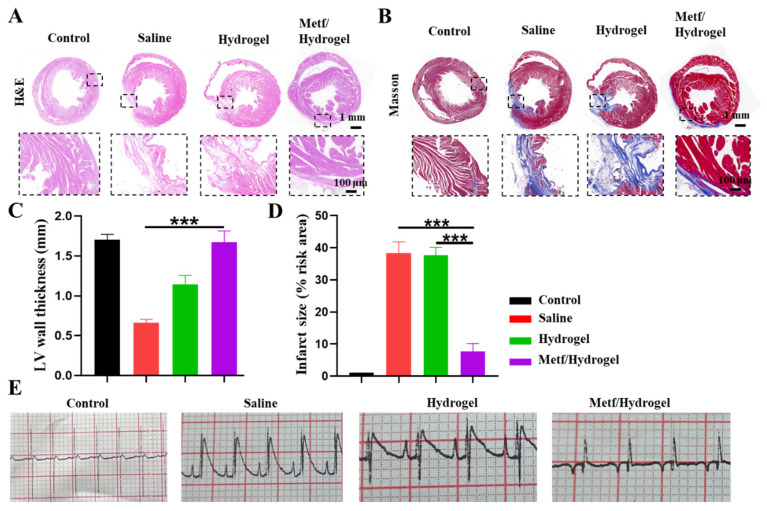
Effects of metf/hydrogel treatment on heart in rats with MI after 28 days of treatment. (**A**) H&E staining of MI in all groups. (**B**) Masson staining of infarcted area in all groups. (**C**,**D**) Quantitative analysis of myocardial wall thickness and infarct area in all groups. (**E**) Representative ECGs of all groups after 4 weeks of treatment (n = 3, *** *p* < 0.001).

After 28 days of treatment, MI areas in all groups were stained with H&E and Masson. The findings indicated that the saline group displayed myocardial wall collapse and notable myocardial fibrosis. In contrast, the metf/hydrogel group substantially inhibited infarct expansion and reduced the collagen area compared with the blank hydrogel group (Figure 5A–D).

Conductive hydrogel, known for its excellent electrical conductivity and biocompatibility, offers unique benefits in treating MI. It enhances the conduction of electrical signals in the myocardium, aiding in restoring normal heart rhythm and function. Electrocardiograms (ECGs) were conducted on SD rats from all groups at the 4-week post-treatment mark using an electrocardiographic apparatus (Figure 5E). The results indicated that the saline group displayed more prominent pathological Q waves and ST-segment elevation, suggesting fibrosis in the infarcted tissue and myocardial atrophy. In contrast, treatment with metf/conductive hydrogel significantly reduced pathological Q waves and prolonged QRS intervals caused by MI compared to the saline group. Notably, the blank conductive hydrogel did not effectively alleviate the pathological features of MI, possibly due to a lack of therapeutic effect or hydrogel degradation.

**Figure 6:**
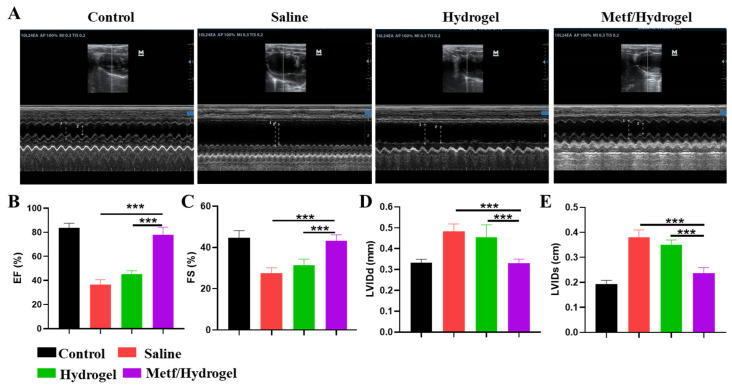
Effect of metf/hydrogel on cardiac function in MI rats. (**A**) ECHO was performed on SD rats from different treatment groups after 4 weeks of treatment. (**B**–**E**) LVEF, LVFS, LVIDd, and LVIDs obtained from ECHO. (n = 3, *** *p* < 0.001).

Following a 4-week treatment, cardiac performance was evaluated using echocardiography (ECHO) across all groups (Figure 6A–E). Both the saline and blank hydrogel groups exhibited typical symptoms of myocardial ischemia, including decreased left ventricular ejection fraction (LVEF) and fractional shortening (FS), as well as increased left ventricular end-systolic volume (LVESV) and end-diastolic volume (LVEDV). In contrast, the metf/hydrogel group showed enhancements in LVEF and LVFS, accompanied by reductions in LVESV and LVEDV, indicating improved cardiac output and ventricular capacity.

## 4. Conclusions

In this study, we developed a conductive CSMA/Pam-GelMA hydrogel system loaded with metf to address heart rate imbalance and mitochondrial damage following MI. The optimized hydrogel formulation demonstrated improved injectability, mechanical strength, and conductivity. Experiments conducted in a controlled environment showed that the hydrogel system’s release of metf improved CM mitochondrial function, reduced mitochondrial oxidative damage, and increased CM activity. Furthermore, in vivo findings revealed that intramyocardial injections of the hydrogel system into rat myocardium post-MI significantly mitigated tissue damage, lowered ROS levels, hindered myocardial fibrosis, and improved cardiac function. This readily prepared conductive metf/hydrogel system shows promising potential for MI therapy.

## Data Availability

The original contributions presented in the study are included in the article, and further inquiries can be directed to the corresponding authors.
